# Supplementary material for: Molecular basis of the TRAP complex function in ER protein biogenesis
Source: Nat Struct Mol Biol. 2023 May 11;30(6):770–7. doi: 10.1038/s41594-023-00990-0 (PMC10279528; doi:10.1038/s41594-023-00990-0)
Supplement: Supplementary file 1 — Supplementary Tables 1–4. [file 41594_2023_990_MOESM1_ESM.pdf]

nature structural & molecular biology

Article https://doi.org/10.1038/s41594-023-00990-0

# Molecular basis of the TRAP complex function in ER protein biogenesis

In the format provided by the authors and unedited

**Supplementary Information Table 1**

| **Sequence of synthetic RNAi-resistant wildtype TRAPα gene (*syn-trap-1*)** |
|---|
| ATGAAGCTTTCCACCGTCTTCCTCCTTGCTGCCCTCGGATTCTGCGCCGTCTACGCT<br>GCTGATGTTGTCGATGGAGAGGTCACCGACGATGCTCCAAAGAACTCTCAAGAGG<br>ATGACGACCTTACCATCGGAGCTTCCCCAGACGCTGGACTCGCTTTCCACTTCGTC<br>CAACCATCCGACGCTAACGTCGTCCGTGAATTCTACACTGGAAAGCCAGTTAAGTA<br>CCTTATCGGATTCCAGgtaagtttaaacatatatatactaactaaccctgattatttaaattttcagAACAAGGGAGA<br>GAAGGATTTCGTTGTTAAGTACGCCGAGACCTCCTTCCGCTTCCCAACCGACCACT<br>CTTACCACCTTCAAAACTTCACCCGTGGAGAGTACAACCGTCGTGTTGCTCCAAAG<br>gtaagtttaaacagttcggtactaactaaccatacatatttaaattttcagGAGGAGGTTACCCTTGATTACGGATT<br>CTACGCTCACGAAACCTTCGCTGGACGTCCAGTCGGACTTGTTGTTAACGTCCACT<br>ACCAAGATGCTGATGGAAACGTCTTCGTTAACAACGTTTACAACCAAACTATCAAC<br>ATCATGGAGGATGATTCCGGATTCTCCGGAGAGACTGGATTCCTTTTCATTTTCTTC<br>GTCGCTCTTTCCATCGGAGGACTTTACCTCTCTAACCAATTCCTCTCTAAGCTTTCT<br>CGTAAGTCCGGACTTTCCAAGCGTCGTGTCGTCGAGCAAGGAACCTCTTCTGAGGT<br>TGATTTCGAGTGGATCCCACGCGACGCTGTTAAGgtaagtttaaacatgattttactaactaactaatctgat<br>ttaaattttcagAACAAGGAGAAGCGCTCTCCAGCTACCGTTTCTCCAAAGGCCCGTAAGT<br>CCGCTAAGAAGGCCGACGATTATAAAGATGATGATGATAAATAA |

Lower case = synthetic introns. Grey = FLAG tag.

**Supplementary Information Table 2**

| **Sequence of synthetic RNAi-resistant wildtype TRAPγ gene (*syn-trap-3*)** |
| --- |
| ATGGGAAAGCTTACTAAGGAGGAGGAGCTCCTTCTTTCTTCTTACTCTGCTACCTC<br>TTCTACTAAGgtaagtttaaacatatatatactaactaaccctgattatttaaattttcagGGAAATCTTTTCTTCTAC<br>CTTAACGCTCTTATCATCTCCATCGCTCCACTTTACCTTTTCTACGGAGTCCATCAA<br>ATGGAGATCCAAGATTCTCTCGTCGTCTGGGGACTTTCCGCTGTCGGAACCGCTTA<br>CCTTCTTTCCCTTGCTTGCAAGAACCAAAAGTGCCTCCTTAAGCACCAAATCGTTA<br>TGAAGCGTGGATCCGCCGTTGAGCGTGAGATCTCTGGACAATACGCCGCCGATAA<br>GgtaagtttaaacagttcggtactaactaaccatacatatttaaattttcagAAGATGACCGTCAAGGAGAAGGAG<br>GAGCGTGCTCTCTTCCGTAAGgtaagtttaaacatgattttactaactaactaatctgatttaaattttcagAACGAG<br>GTTGCTGATACCGAGTCTACCTACCTTTCTGTTTTCTACACCAACTCTCTTTACCTT<br>ACCATCATGCTTGTCTCCGCTTTCTTCCTTCTTGCTAACGTTGCCCCAGTCTTCAAC<br>CTTCTTATCTCTACCATCGGATCCGCTGGACTTGTCGCTTTCCTTTCCACCGCCAAG<br>AACGATTATAAAGATGATGATGATAAATAA |

Lower case = synthetic introns. Grey = FLAG tag.

**Supplementary Information Table 3**

| | ***C. elegans* strains** |
|---|---|
| DEU156 | gamSi56 [*trap-1p::WT-syn-trap-1-FLAG*; *icd-2p::mCherry*] |
| DEU157 | gamSi57 [*trap-1p::W228A-K239E-K235E-syn-trap-1-FLAG*; *icd-2p::mCherry*] |
| DEU158 | gamSi58 [*trap-1p::*$^{133}$HETFAGR/SSSSSSS$^{139}$*-syn-trap-1-FLAG*; *icd-2p::mCherry*] |
| DEU159 | gamSi59 [*trap-1p::*$^{133}$HETFAGR/SSSSSSS$^{139}$*-W228A-K239E-K235E-syn-trap-1-FLAG*; *icd-2p::mCherry*] |
| DEU160 | gamSi60 [*trap-1p::F98T-Y104T-L106T-F109T-L126T-Y128T-Y131T-V141T-syn-trap-1-FLAG*; *icd-2p::mCherry*] |
| DEU161 | gamSi61 [*trap-3p::WT-syn-trap-3-FLAG*; *icd-2p::mCherry*] |
| DEU162 | gamSi62 [*trap-3p::K103E-K104E-K108E-syn-trap-3-FLAG*; *icd-2p::mCherry*] |
| DEU163 | trap-1(gk5960); zcIs4 [*hsp-4p::GFP*; *loxP* + *myo-2p::GFP* + *rps-27p::neoR* + *loxP*] |
| DEU164 | trap-1(gk5960); zcIs4; gamSi56 [*hsp-4p::GFP*; *loxP* + *myo-2p::GFP* + *rps-27p::neoR* + *loxP; trap-1p::WT-syn-trap-1-FLAG; icd-2p::mCherry*] |
| DEU165 | trap-1(gk5960); zcIs4; gamSi57 [*hsp-4p::GFP*; *loxP* + *myo-2p::GFP* + *rps-27p::neoR* + *loxP; trap-1p::W228A-K239E-K235E-syn-trap-1-FLAG; icd-2p::mCherry*] |
| DEU166 | trap-1(gk5960); zcIs4; gamSi58 [*hsp-4p::GFP*; *loxP* + *myo-2p::GFP* + *rps-27p::neoR* + *loxP; trap-1p:* $^{133}$HETFAGR/SSSSSSS$^{139}$*-syn-trap-1-FLAG; icd-2p::mCherry*] |
| DEU167 | trap-1(gk5960); zcIs4; gamSi59 [*hsp-4p::GFP*; *loxP* + *myo-2p::GFP* + *rps-27p::neoR* + *loxP; trap-1p::*$^{133}$HETFAGR/SSSSSSS$^{139}$*-W228A-K239E-K235E -syn-trap-1-FLAG; icd-2p::mCherry*] |
| DEU168 | trap-1(gk5960); zcIs4; gamSi60 [*hsp-4p::GFP*; *loxP* + *myo-2p::GFP* + *rps-27p::neoR* + *loxP; trap-1p::F98T-Y104T-L106T-F109T-L126T-Y128T-Y131T-V141T-syn-trap-1-FLAG; icd-2p::mCherry*] |
| DEU169 | zcIs4; gamSi61 [*hsp-4p::GFP*; *trap-3p::WT-syn-trap-3-FLAG*; *icd-2p::mCherry*] |
| DEU170 | zcIs4; gamSi62 [*hsp-4p::GFP*; *trap-3p::K103E-K104E-K108E-syn-trap-3-FLAG*; *icd-2p::mCherry*] |
| DEU171 | zcIs4; gamSi56; gamSi61 [*hsp-4p::GFP*; *trap-1p::WT-syn-trap-1-FLAG; trap-3p::WT-syn-trap-3-FLAG*; *icd-2p::mCherry*] |
| DEU172 | zcIs4; gamSi56; gamSi62 [*hsp-4p::GFP*; *trap-1p::WT-syn-trap-1-FLAG; trap-3p::K103E-K104E-K108E-syn-trap-3-FLAG*; *icd-2p::mCherry*] |
| DEU173 | zcIs4; gamSi57; gamSi61 [*hsp-4p::GFP*; *trap-1p::W228A-K239E-K235E-syn-trap-1-FLAG; trap-3p::WT-syn-trap-3-FLAG*; *icd-2p::mCherry*] |
| DEU174 | zcIs4; gamSi57; gamSi62 [*hsp-4p::GFP*; *trap-1p::W228A-K239E-K235E-syn-trap-1-FLAG; trap-3p::K103E-K104E-K108E-syn-trap-3-FLAG*; *icd-2p::mCherry*] |

| | |
|---|---|
| DEU175 | daf-2(e1368); trap-1(gk5960) [daf-2(e1368) III.; *loxP* + *myo-2p::GFP* + *rps-27p::neoR* + *loxP*] |
| DEU176 | daf-2(e1368); trap-1(gk5960); gamSi56 [daf-2(e1368) III.; *loxP* + *myo-2p::GFP* + *rps-27p::neoR* + *loxP; trap-1p::WT-syn-trap-1-FLAG*; *icd-2p::mCherry*] |
| DEU177 | daf-2(e1368); trap-1(gk5960); gamSi57 [daf-2(e1368) III.; *loxP* + *myo-2p::GFP* + *rps-27p::neoR* + *loxP; trap-1p::W228A-K239E-K235E-syn-trap-1-FLAG*; *icd-2p::mCherry*] |
| DEU178 | daf-2(e1368); trap-1(gk5960); gamSi60 [daf-2(e1368) III.; *loxP* + *myo-2p::GFP* + *rps-27p::neoR* + *loxP; trap-1p::F98T-Y104T-L106T-F109T-L126T-Y128T-Y131T-V141T-syn-trap-1-FLAG*; *icd-2p::mCherry*] |

**Supplementary Information Table 4.** Antibodies used in this study

| Antibody | Source | Catalog No. | RRID | Dilution for blotting |
|---|---|---|---|---|
| Rabbit anti-FLAG | Sigma-Aldrich | F7425 | AB_439687 | 1:2000 |
| Mouse anti-GAPDH | Proteintech | 60004-1-Ig | AB_2107436 | 1.5000 |
| Mouse anti-Tubulin | DSHB | AA4.3 | AB_579793 | 1:500 |
