# Supplementary material for: Molecular basis of the TRAP complex function in ER protein biogenesis
Source: Nat Struct Mol Biol. 2023 May 11;30(6):770–7. doi: 10.1038/s41594-023-00990-0 (PMC10279528; doi:10.1038/s41594-023-00990-0)
Supplement: Supplementary file 2 — Reporting Summary [file 41594_2023_990_MOESM2_ESM.pdf]

nature portfolio

Corresponding author(s): Ahmad Jomaa, Elke Deuerling, Nenad Ban

Last updated by author(s): 3/22/2023

Reporting Summary

Nature Portfolio wishes to improve the reproducibility of the work that we publish. This form provides structure for consistency and transparency in reporting. For further information on Nature Portfolio policies, see our Editorial Policies and the Editorial Policy Checklist.

Statistics

For all statistical analyses, confirm that the following items are present in the figure legend, table legend, main text, or Methods section.

n/a	Confirmed	
☐	☒	The exact sample size (*n*) for each experimental group/condition, given as a discrete number and unit of measurement
☒	☐	A statement on whether measurements were taken from distinct samples or whether the same sample was measured repeatedly
☒	☐	The statistical test(s) used AND whether they are one- or two-sided<br>*Only common tests should be described solely by name; describe more complex techniques in the Methods section.*
☒	☐	A description of all covariates tested
☒	☐	A description of any assumptions or corrections, such as tests of normality and adjustment for multiple comparisons
☐	☒	A full description of the statistical parameters including central tendency (e.g. means) or other basic estimates (e.g. regression coefficient) AND variation (e.g. standard deviation) or associated estimates of uncertainty (e.g. confidence intervals)
☒	☐	For null hypothesis testing, the test statistic (e.g. *F*, *t*, *r*) with confidence intervals, effect sizes, degrees of freedom and *P* value noted<br>*Give P values as exact values whenever suitable.*
☒	☐	For Bayesian analysis, information on the choice of priors and Markov chain Monte Carlo settings
☒	☐	For hierarchical and complex designs, identification of the appropriate level for tests and full reporting of outcomes
☒	☐	Estimates of effect sizes (e.g. Cohen's *d*, Pearson's *r*), indicating how they were calculated

Our web collection on statistics for biologists contains articles on many of the points above.

Software and code

Policy information about availability of computer code

Data collection	EPU 2 (Thermo Fisher)
Data analysis	RELION 3.1<br>MotionCorr2<br>Coot 0.9.8.1<br>Phenix 1.20.1<br>UCSF Chimera 1.15<br>UCSF ChimeraX 1.1.1<br>MolProbity<br>AlphaFold2

For manuscripts utilizing custom algorithms or software that are central to the research but not yet described in published literature, software must be made available to editors and reviewers. We strongly encourage code deposition in a community repository (e.g. GitHub). See the Nature Portfolio guidelines for submitting code & software for further information.

Data

Policy information about availability of data

All manuscripts must include a data availability statement. This statement should provide the following information, where applicable:

- Accession codes, unique identifiers, or web links for publicly available datasets
- A description of any restrictions on data availability
- For clinical datasets or third party data, please ensure that the statement adheres to our policy

The data supporting the findings of this study are available in the Electron Microscopy Bank and Protein Data Bank under accession codes EMD-16232 and PDB ID: 8BTK. The structures of rabbit 80S ribosome (PDB 7O7Y) and Sec61 translocon (PDB 6W6L) were used for comparisons and as an initial model. Source data are provided with the manuscript.

Human research participants

Policy information about studies involving human research participants and Sex and Gender in Research.

Reporting on sex and gender	Not relevant to this study.
Population characteristics	Not relevant to this study.
Recruitment	Not relevant to this study.
Ethics oversight	Not relevant to this study.

Note that full information on the approval of the study protocol must also be provided in the manuscript.

Field-specific reporting

Please select the one below that is the best fit for your research. If you are not sure, read the appropriate sections before making your selection.

☒ Life sciences ☐ Behavioural & social sciences ☐ Ecological, evolutionary & environmental sciences

For a reference copy of the document with all sections, see nature.com/documents/nr-reporting-summary-flat.pdf

Life sciences study design

All studies must disclose on these points even when the disclosure is negative.

Sample size	No sample size calculations were peformed. The sample size for the worm growth analysis was determined by the number of eggs that were laid during the synchronization step.
Data exclusions	No data were excluded from the analyses.
Replication	Each worm experiment was independently repeated three times with similar results.
Randomization	Samples were not randomized because there is nothing to randomize. The different conditions being compared within any given experiment derive from a single common stock.
Blinding	Not relevant to this study. The data were determined by technical means and not by human judgment.

Reporting for specific materials, systems and methods

We require information from authors about some types of materials, experimental systems and methods used in many studies. Here, indicate whether each material, system or method listed is relevant to your study. If you are not sure if a list item applies to your research, read the appropriate section before selecting a response.

Materials & experimental systems

n/a	Involved in the study
☐	☒ Antibodies
☒	☐ Eukaryotic cell lines
☒	☐ Palaeontology and archaeology
☐	☒ Animals and other organisms
☒	☐ Clinical data
☒	☐ Dual use research of concern

Methods

n/a	Involved in the study
☒	☐ ChIP-seq
☒	☐ Flow cytometry
☒	☐ MRI-based neuroimaging

Antibodies

Antibodies used	FLAG (Sigma, #F7425), GAPDH (Proteintech, #60004-1-lg), Tubulin (DSHB, clone AA4.3). A table of all antibodies used in this study, their catalog number, and specific conditions for use, are provided in Supplementary Table 1.
Validation	Each antibody was validated for specificity against the antigen in immunoblot analyses by the manufacturerer. FLAG (Sigma, #F7425) and GAPDH (Proteintech, #60004-1-lg) antibodies were used in over 100 different publications, and the Tubulin (DSHB, clone AA4.3) antibody in 39 publications, according to Scicrunch.org.

Animals and other research organisms

Policy information about studies involving animals; ARRIVE guidelines recommended for reporting animal research, and Sex and Gender in Research

Laboratory animals	Caenorhabditis elegans (strain N2, strain SJ4005, strain VC4892, strain DR1572). Animals were studied in the larval and adult day stage.
Wild animals	This study did not involve wild animals.
Reporting on sex	Not relevant to this study. Only hermaphrodites were analyzed.
Field-collected samples	This study did not involve samples collected from the field.
Ethics oversight	Not relevant to this study.

Note that full information on the approval of the study protocol must also be provided in the manuscript.
