# Supplementary figures and images for: Molecular basis of the TRAP complex function in ER protein biogenesis
Source: Nat Struct Mol Biol. 2023 May 11;30(6):770–7. doi: 10.1038/s41594-023-00990-0 (PMC10279528; doi:10.1038/s41594-023-00990-0)

Source Data – Extended Data Figure 9. Uncropped blots.

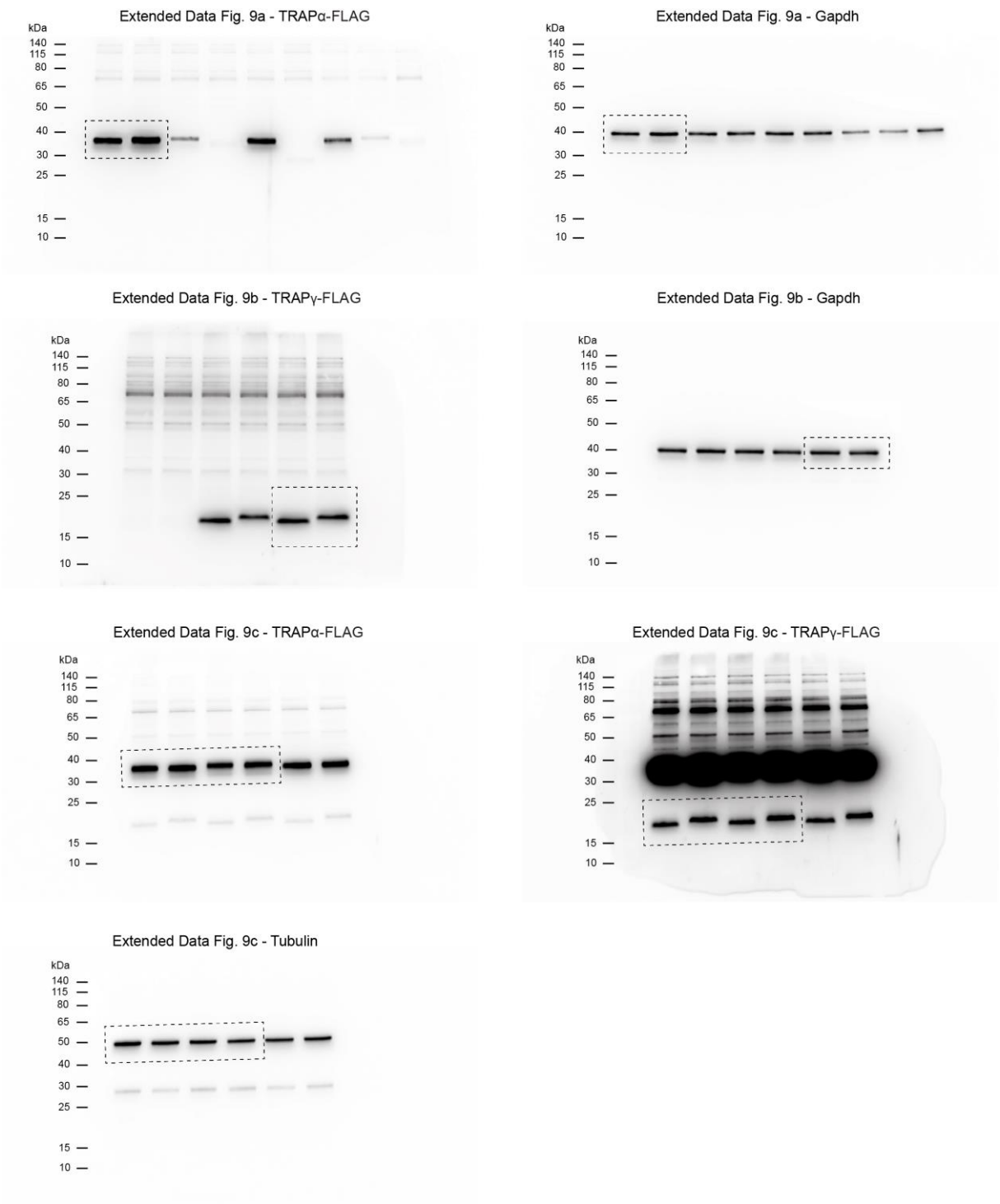

Supplement: Source Data Extended Data Fig. 9 — Uncropped blots. [file 41594_2023_990_MOESM4_ESM.pdf]

Source Data – Extended Data Figure 10. Uncropped blots.

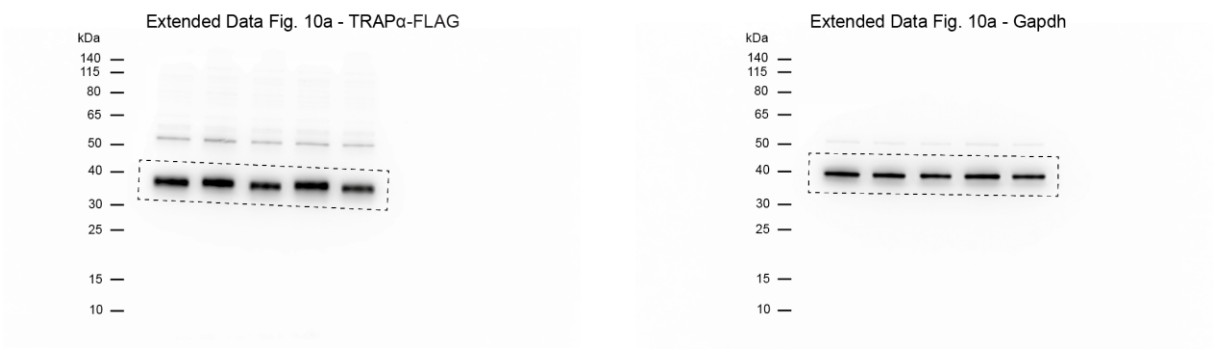

Supplement: Source Data Extended Data Fig. 10 — Uncropped blots. [file 41594_2023_990_MOESM5_ESM.pdf]
